# Metal-Regulatory Transcription Factor-1 Targeted by miR-148a-3p Is Implicated in Human Hepatocellular Carcinoma Progression

**DOI:** 10.3389/fonc.2021.700649

**Published:** 2021-09-29

**Authors:** Zhuozhen Lyu, Mingyu Yang, Tan Yang, Mingze Ma, Zhen Yang

**Affiliations:** ^1^ Department of Infectious Diseases, Shandong Provincial Hospital Affiliated to Shandong First Medical University, Jinan, China; ^2^ Department of Infectious Diseases, Jining First People’s Hospital, Jining, China

**Keywords:** hepatocellular carcinoma, copper, MTF-1, APE/Ref-1, miR-148a-3p

## Abstract

Metal-regulatory transcription factor-1 (MTF-1) is of importance in maintaining metal homeostasis. Copper exposure considerably stimulates the proliferation of hepatocellular carcinoma (HCC) cells with enhanced MTF-1 expression. However, the underlying molecular mechanisms have not been completely elucidated. In this study, we utilized different approaches to investigate the potential role of MTF-1 involved in HCC progression. The expression levels of MTF-1 and miR-148a-3p were determined using real-time polymerase chain reaction (PCR), Western blotting, and immunohistochemistry. The interaction of MTF-1 with apurinic apyrimidinic endonuclease/redox effector factor 1 (APE/Ref-1) or miR-148a-3p was determined using immunoprecipitation or dual-luciferase reporter assay, respectively. Cell viability and metastatic ability were evaluated using colony formation, 3-(4,5-dimethylthiazol-2-yl)-2,5-diphenyltetrazolium bromide (MTT), wound scratch, and Transwell assays, and apoptotic cells were detected by flow cytometry. The biological functions of MTF-1 and miR-148a-3p were also determined using a xenograft mouse model. MTF-1 expression was upregulated in HCC cells and was associated with poor survival and recurrence. MTF-1 overexpression enhanced the proliferation and metastatic potential of HCC cells. Further mechanistic analyses demonstrated that MTF-1 bound to APE/Ref-1 and that MTF-1 is a direct target of miR-148-3p, which inversely regulated MTF-1 transcription activity. MiR-148a-3p overexpression effectively inhibited HCC cell proliferation and metastasis stimulated by MTF-1, with increased apoptosis. There was a decrease in miR-148a-3p expression in exosomes isolated from the plasma of patients with HCC and HCC cell culture supernatants. Co-incubation of HCC cells with exosomes from hepatocyte-conditioned media inhibited cell migration and caused apoptosis. The *in vivo* study revealed slow growth of MTF-1-knockdown and miR-148a-3p-overexpressing Hep3B-derived xenografts, with reduced tumor volume and weight compared with the control group. Collectively, these findings implicate MTF-1 as a modulator of HCC tumorigenesis and progression. Selective targeting towards exosomal miR-148a-3p, which might contribute to the negative regulation of MTF-1 at least partially in HCC, demonstrates therapeutic benefits for patients with HCC.

## Introduction

Hepatocellular carcinoma (HCC) is an aggressive neoplasm with a 5-year survival rate of only 15% (1). The high mortality rate is attributed to late-stage detection of this cancer, when most of the available therapies are not effective. HCC has been projected to be the third important cause of cancer-related deaths by 2030 (2). Therefore, a deeper understanding of the mechanisms contributing to HCC progression is crucial to investigate the potential therapeutic targets.

Metal, particularly copper, has been shown to play a crucial role in HCC genesis and progression (3, 4). Copper level, which is associated with HCC prognosis, increases in the serum and tumor biopsies of patients with HCC (5). Excess copper is deleterious for cell metabolism. Hence, copper level in cells and tissue must be closely regulated. Metal-regulatory transcription factor-1 (MTF-1) is a transcription factor that maintains metal homeostasis and protects cells against injury by excess metals, including copper. In recent years, several studies have focused on the roles of MTF-1 in tumorigenesis and disease progression. Upregulated MTF-1 level is detected in breast cancer, lung carcinoma, and cholangiocarcinoma (6, 7). In our preliminary study, we found that MTF-1 expression, which is copper inducible, was upregulated in HCC cells and biopsies of patients with HCC. However, the role of MTF-1 in HCC genesis and progression and the underlying mechanisms have not been well defined.

Exosomes are extracellular vesicles of diameters ranging from 30 to 150 nm and are present in various body fluids. MicroRNAs (miRNAs), a major class of small non-coding RNAs that mediate post-transcriptional gene silencing by binding to the 3′-untranslated region (UTR) or open reading frames (ORFs) of target mRNAs, have been identified in exosomes (8). Accumulating evidence has indicated that exosomal miRNAs from cancer cells mediate tumor metastasis and chemoresistance (9, 10). miR-148a-3p, a member of the miR-148/152 family, functions as a tumor suppressor (11–13). In our preliminary study, we found that miR-148a-3p expression decreased in the exosomes isolated from the plasma of patients with HCC and HCC cell culture media. Moreover, further analysis indicated that MTF-1 might be a direct target of miR-148a-3p. Thus, the primary objective of this study was to investigate the functional role of MTF-1 in HCC. Furthermore, miR-148-3p-containing exosomes inhibited HCC cell proliferation and metastasis by inhibiting MTF-1, suggesting that further exploration targeting MTF-1 would be of importance for HCC treatment.

## Materials and Methods

### Cell Culture

Immortalized non-malignant hepatocyte cell line (THLE3) and human embryonic kidney 293T cell line were purchased from ZQXZ Biotechnology Company (Shanghai, China). Human HCC cell lines (Hep3B and SNU-387) were purchased from the Procell Life Science Technology Company (Wuhan, China) with STR certificate. They were all cultured in medium supplemented with 10% FBS, 100 U/ml penicillin G, and 100 μg/ml streptomycin sulfate, in 5% CO_2_-humidified atmosphere, at 37°C. The passage numbers for Hep3B and SNU-387 used in these experiments were ≤8 and ≤5 for HEK 293T and THLE3, respectively.

### Patients

Ten patients with HCC were enrolled from Shandong Provincial Hospital. The study was approved by the Institutional Medical Ethics Committee of Shandong Provincial Hospital. The inclusion criteria were as follows: at least 18 years old and diagnosed with HCC without ongoing active treatments. The exclusion criteria were as follows: patients with other types of cancer, renal or liver failure, and taking medications that affect liver function. All subjects signed an Institutional Review Board (IRB)-approved informed consent before enrollment in the study. Blood samples were collected from patients before surgery or before any local or systemic treatment to obtain plasma for further analyses.

### Immunohistochemistry and Scoring

Unstained human HCC tissue microarrays (TMAs) were purchased from Shanghai Outdo Biotech Co., Ltd. (Shanghai, China), and used according to the manufacturer’s instructions. The TMAs were first incubated with the primary antibody and then with the secondary antibody. The Envision and DAB kit (Santa Cruz Biotechnology, Dallas, TX, United States) was used to visualize and assess the TMAs. The slides were imaged using a microscope slide scanner (Pannoramic DESK, 3DHISTECH, Budapest, Hungary) and analyzed using QuantCenter 2.1 (3DHISTECH, Budapest, Hungary). Immunohistochemical localization was scored in a semi-quantitative fashion incorporating both intensity and distribution of specific staining. The intensity of specific staining was characterized as not present (0), weak but detectable above control (1+), moderate (2+), and very strong (3+). Histochemistry score (H-score) = ∑(pi × i) = (percentage of weak intensity cells ×1) + (percentage of moderate intensity cells ×2) + (percentage of strong intensity cells ×3), where pi was the positive cell percentage and i represents the intensity of staining ranging from 1 to 3. The median of log_2_ H-score was defined as a cutoff value, based on which the data were divided into high and low groups. Next, the clinical characteristics and outcomes were analyzed and compared. The representative images were captured and processed. Results with *p* < 0.05 were considered statistically significant.

### Fluorescence *In Situ* Hybridization Analysis

Unstained human HCC TMAs were used in the analysis. Briefly, the TMAs were dewaxed in xylene and rehydrated through a gradient series of ethanol. After pretreatment, the tissue sections were subjected to fluorescence *in situ* hybridization (FISH). miR-148a-3p probe (5′-ACTTCTATCATACTCAGAGTCGGAGTGTCT-3′), 5′-(ttt CATCATCAT ACATCATCAT)_30_-3′, and 5′-DIG-tt ATGATGATGTATGATGATGT-3′ were obtained from Servicebio (Wuhan, China). The average positive area (µm^2^) was defined using HALOImage analysis software. Data are presented as mean ± SD.

### Cell Protein Extraction and Western Blotting

Cells were collected and lysed in the Cell Lysis Buffer (Beyotime, Shanghai, China) for the analysis. The protein concentration was measured using the bicinchoninic acid (BCA) Protein Assay Kit (Beyotime, Shanghai, China). Samples were then subjected to sodium dodecyl sulfate/polyacrylamide gel electrophoresis (SDS/PAGE, Servicebio, Wuhan, China) and quantified by band densitometry (Tanon5200, Shanghai, China).

The following primary antibodies were used for Western blotting and IHC: anti-MTF-1, anti-APE/Ref-1 antibody (Santa Cruz Biotechnology, Santa Cruz, CA, USA), anti-calnexin, anti-TSG101, and anti-CD63 antibodies (Abcam, Shanghai, China). GAPDH and α-tubulin were used as internal references (Sigma Life Sciences, St. Louis, MO, USA). The following secondary antibodies were obtained from ZSGB-Bio (Beijing, China): horseradish peroxidase (HRP)-conjugated anti-mouse antibody and anti-rabbit antibody.

### Real-Time Reverse Transcription-Polymerase Chain Reaction

The total RNA was isolated using the Ultrapure RNA Kit (CWBIO, Beijing, China), according to the manufacturer’s protocol. Equal amounts of samples were amplified. Real-time reverse transcription-polymerase chain reaction (RT-PCR) was performed with the SYBR Green PCR Premix HS-Taq kit (AG Biology, Changsha, Hunan, China) and detected using Tanon5200 (Shanghai, China). The following primer pairs were used: MTF-1, forward: 5’-GCTGGGGAGGGGAGAAGC-3’, reverse: 5’-ACTGTGTTCCCCCCATGGTTC-3’; MiR-148a-3p, forward: 5’-GGAAAGTTCTGAGACACTC-3’, reverse: 5’-CAGTGCGTGTCGTGGAGT-3’; APE/Ref-1, forward: 5’-ACTGTGCCTTCAAGAGACCAA-3’, reverse: 5’-TCATCGCCTATGCCGTAAGAA-3’ (DingGuo Biotechnology, Beijing, China). Data were analyzed using the comparative C*t* method; 2^−ΔΔCt^ showed the difference between the treatments and control.

### Transfection Assay

Overexpression plasmids of MTF-1 and APE/Ref-1 and the control empty vector were obtained from ViGene Biosciences (Jinan, Shandong, China). The shRNA targeting human MTF-1 was obtained from GenePharma (Shanghai, China). MiR-148a-3p mimic, miR-148a-3p inhibitor, and their respective control were synthesized at RIBOBIO (Guangzhou, China). Hsa-miR-148a-3p mimic: sense: 5’-UCAGUGCACUACAGAACUUUGU-3’, antisense: 5’-ACAAAGUUCUGUAGUGCACUGA-3’; hsa-miR-148a-3p mimic control: sense: 5’-UUUGUACUACACAAAAGUACUG-3’, antisense: 5’-CAGUACUUUUGUGUAGUACAAA-3’; hsa-miR-148a-3p inhibitor: 5’-ACAAAGUUCUGUAGUGCACUGA-3’; and hsa-miR-148a-3p inhibitor control: 5’-CAGUACUUUUGUGUAGUACAAA-3’ were used. Hep3B cells either with MTF-1 stable knockdown or with miR-148a-3p stable overexpression were mediated by lentivirus infection for *in vivo* studies. The cells in six-well plates were transfected using Lipofectamine 2000 (Invitrogen Inc., Carlsbad, CA, USA), according to the manufacturer’s instructions. Serum-free medium was replaced with regular growth medium 6 h after transfection, and subsequent experiments were carried out.

### Luciferase Reporter Assay

The sequence containing three predicted binding sites with miR-148a-3p or mutant variant in the 3′-UTR was cloned into the psiCHECK™-2-vector (Promega, Madison, WI, USA). Approximately 2 × 10^4^ HEK 293T cells/well were seeded and the recombinant plasmids were co-transfected into cells with either negative control or miR-148a-3p mimic using Lipofectamine 2000 (Invitrogen Inc.). After 48 h, the luciferase activity was performed using the Dual-Luciferase^®^ Reporter 1000 Assay System (Promega, Madison, WI, USA), according to the manufacturer’s protocol. Firefly luciferase level was normalized against *Renilla* luciferase activity, and the ratio of firefly/*Renilla* luciferase activity is presented. Experiments were independently performed in triplicate.

### Cell Viability Assay

Cell viability before and after various treatments was determined using the 3-(4,5-dimethylthiazol-2-yl)-2,5-diphenyltetrazolium bromide (MTT) assay. Briefly, the cells (approximately 1,500 cells/well) were seeded in 96-well plates before treatments. At the end of each treatment, MTT reagent was added to the medium. The cells were incubated for an additional 4 h, and the absorbance of the samples was measured at 490 nm using a plate reader (Thermo Fisher, MA, USA). Experiments were performed in triplicate; data are expressed as mean of optical density ± SD.

### Colony Formation Assay

Cells (approximately 1,000 cells/well) were seeded in a six-well plate either with or after different treatments. After 14 days, the cells were fixed with 4% paraformaldehyde and stained with 0.5% crystal violet. Individual clones (>50 cells/clone) were counted. Each treatment was performed in triplicate. Data are expressed as mean ± SD.

### Wound Healing Assay

Cells (approximately 10^6^ cells/well) were cultured in a six-well plate for 24 h; the cell layer was scratched with a sterile glass tip and cultured in medium supplemented with 10% fetal bovine serum (FBS) for up to 48 h. Cell images were captured under an Olympic microscope (CKX53; Olympus, Tokyo, Japan); data from triplicate assays were analyzed.

### Migration and Invasion Assays

Approximately 1 × 10^5^ cells/well in a single cell suspension were added to the upper chamber of a 24-well plate. Medium (600 μl) with 20% FBS was added into the bottom chamber. After migration through the Transwell membrane, the cells were fixed with 4% paraformaldehyde and stained with crystal violet (Sangon Biotech). The cells were then counted under the microscope (CKX53; Olympus) at 100×. The difference between the migration and invasion assays was that the Transwell chambers for migration assays were not coated with Matrigel. All Transwell treatments were conducted in triplicate.

### Flow Cytometry

Following different treatments, the cells were collected and washed once with 1× PBS and fixed in cold 70% ethanol until use. The cells were incubated in propidium iodide staining solution in the dark for 30 min. Ten thousand total events were counted for each sample, which was analyzed for apoptosis using the Annexin V-FITC/7-AAD apoptosis detection kit (Meilunbio, Dalian, Liaoning, China), according to the manufacturer’s protocol.

### Exosome Isolation and Characterization

Cells grown to 70%–80% confluence were washed twice with 1× PBS and then grown in serum-free medium. After 48 h, the conditioned medium was collected and centrifuged at 300 × *g* for 10 min, 2000 × *g* for 10 min, and 10,000 × *g* for 30 min at 4°C to thoroughly remove cellular debris. The supernatants were recentrifuged at 100,000 × *g* for 70 min at 4°C. The pellets were washed, ultra-centrifuged, and re-suspended in 1× PBS. Plasma samples were centrifuged using the same method to collect exosome samples.

The morphology of exosomes was observed by transmission electron microscopy. Briefly, 10 μl of the medium was placed on formvar/carbon-coated copper grids and then stained with 3% aqueous phosphotungstic acid for 35 s. Subsequently, the exosomes were examined using a transmission electron microscope (Tecnai 12, Philips, Amsterdam, Netherlands). Size distribution of exosomes was analyzed using the NanoSight LM10 system equipped with a fast video capture and particle-tracking software (NanoSight, Amesbury, UK). Western blotting was conducted to determine exosome-specific markers (calnexin, TSG101, and CD63).

### Exosome Internalization Assay

Exosomes were labeled with PKH-26 red fluorescent dye using the CellLinker Kit (UmiBio, Shanghai, China), according to the manufacturer’s protocol. The labeled exosomes were co-cultured with HCC cells for 30 h at 37°C. The uptake of labeled exosomes by the recipient cells was observed using a Nikon Eclipse fluorescence microscope (Nikon, Tokyo, Japan).

### HCC Tumor-Xenograft Mouse Model

The animal study was approved by the Animal Ethics Committee of Shandong Provincial Hospital; 25-week-old BALB/c nude mice were purchased from Beijing Vital River Laboratory Animal Technology Co., Ltd. and maintained under specific pathogen-free conditions. After different treatments, human Hep3B cells (approximately 1 × 10^7^ cells per mice) were subcutaneously injected into the mice (*n* = 6 per treatment group). Tumor size was measured weekly and tumor volume was calculated using the following formula: volume (mm^3^) = tumor length × width^2^/2. Six weeks later, all mice were sacrificed to compare the tumor volume and weight.

### Statistical Analysis

Data are presented in bar plots as mean ± SD of at least three independent experiments. All *p*-values were determined using two-sided tests. Correlations among MTF-1, miR-148a-3p, and APE/Ref-1 were assessed using R Hmisc package. Association between MTF-1 immunostaining and clinical characteristics was evaluated using R v3.6.1. Wilcoxon test for two groups and Kruskal–Wallis test for more than two groups were used to verify the correlations. Univariate Cox regression and survival analyses were also conducted. Results with *p* < 0.05 were considered statistically significant.

## Results

### MTF-1 Expression Is Upregulated in HCC

**Figure 1:**
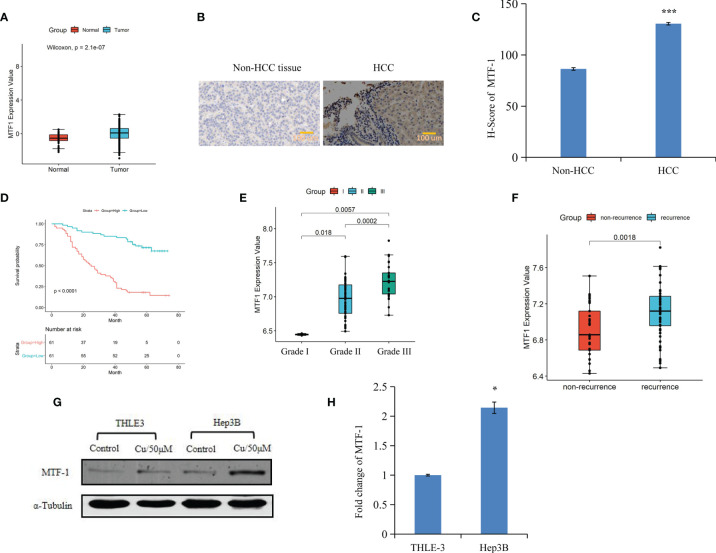
Metal-regulatory transcription factor-1 (MTF-1) is upregulated in hepatocellular carcinoma (HCC). MTF-1 expression was assessed using an integrative analysis of The Cancer Genome Atlas (TCGA) data **(A)**. Human tissue microarray was subjected to immunohistochemistry (IHC). Representative images are shown. Scale bar = 100 μm **(B)**. H-score of MTF-1 in HCC was higher than that in paracancer tissue **(C)**. Higher level of MTF-1 was associated with poor survival **(D)**. MTF-1 level was found to be elevated in cases of poorly differentiated HCC (*p* < 0.01) **(E)** and in recurrence (*p* < 0.005) **(F)**. Strong expression of MTF-1 in HCC cells was determined by Western blotting and RT-PCR **(G, H)**. Data are expressed as mean ± SD of three independent experiments. *p < 0.05, ***p < 0.001.

An integrative analysis of The Cancer Genome Atlas (TCGA) data revealed an increase in MTF-1 expression in tumors than that of normal tissue (**Figure 1A**). To delineate the role of MTF-1 in HCC, we examined tissues by IHC. Enhanced MTF-1 expression was demonstrated in HCC compared with that in paracancer tissue (**Figure 1B**). The H-score of MTF-1 in HCC was evidently increased (**Figure 1C**), and higher MTF-1 expression was associated with poor survival (**Figure 1D**). The correlation analysis of age, sex, tumor characteristics (tumor size, presence of a capsule, stage, grade, and recurrence), and MTF-1 was performed. MTF-1 expression was upregulated in cases with poorly differentiated HCC (*p* < 0.01) (**Figure 1E**) and with recurrence (*p* < 0.005) (**Figure 1F**). However, there was no correlation among age, sex, tumor size, presence of a capsule, and stage (**Supplementary Figures S1A–E**).

Cox proportional hazards model analysis was performed to determine the relationship between clinical variables and the overall survival (OS) of patients with HCC. As shown in **Table 1**, MTF-1, stage, recurrence, and alpha-fetoprotein (AFP) level significantly correlated with OS. In addition, Western blotting and RT-PCR analysis revealed higher MTF-1 expression in HCC cells compared with that in THLE3 cells, especially upon stimulation with copper (**Figures 1G, H**). These findings suggested that MTF-1 upregulation is closely associated with the clinical outcome of patients with HCC and might be an independent risk factor of HCC recurrence and patient mortality.

**Table 1:** Univariate analysis of MTF-1 and clinical parameters with overall survival.

Parameter	Univariate analysis	*p*
	HR (95% CI)	
Metal-regulatory transcription factor-1 (MTF-1)	3.796 (1.614–8.929)	0.002
Sex	1.655 (0.743–3.686)	0.218
Age, years	1.276 (0.686–2.376)	0.442
Grade		
II	25987640.69 (0-0-Inf-Inf)	0.997
III	24252766.538 (0-0-Inf-Inf)	0.997
Stage	1.972 (1.115–3.486)	0.019
Tumor size (cm)	1.243 (0.677–2.283)	0.483
Tumor capsule	1.013 (0.578–1.777)	0.963
Recurrence	6.526 (2.914–14.614)	< 0.001
AFP (μg/L)	1 (1–1)	0.003

### MTF-1 Promotes Cell Proliferation, Migration, and Invasion in HCC

**Figure 2:**
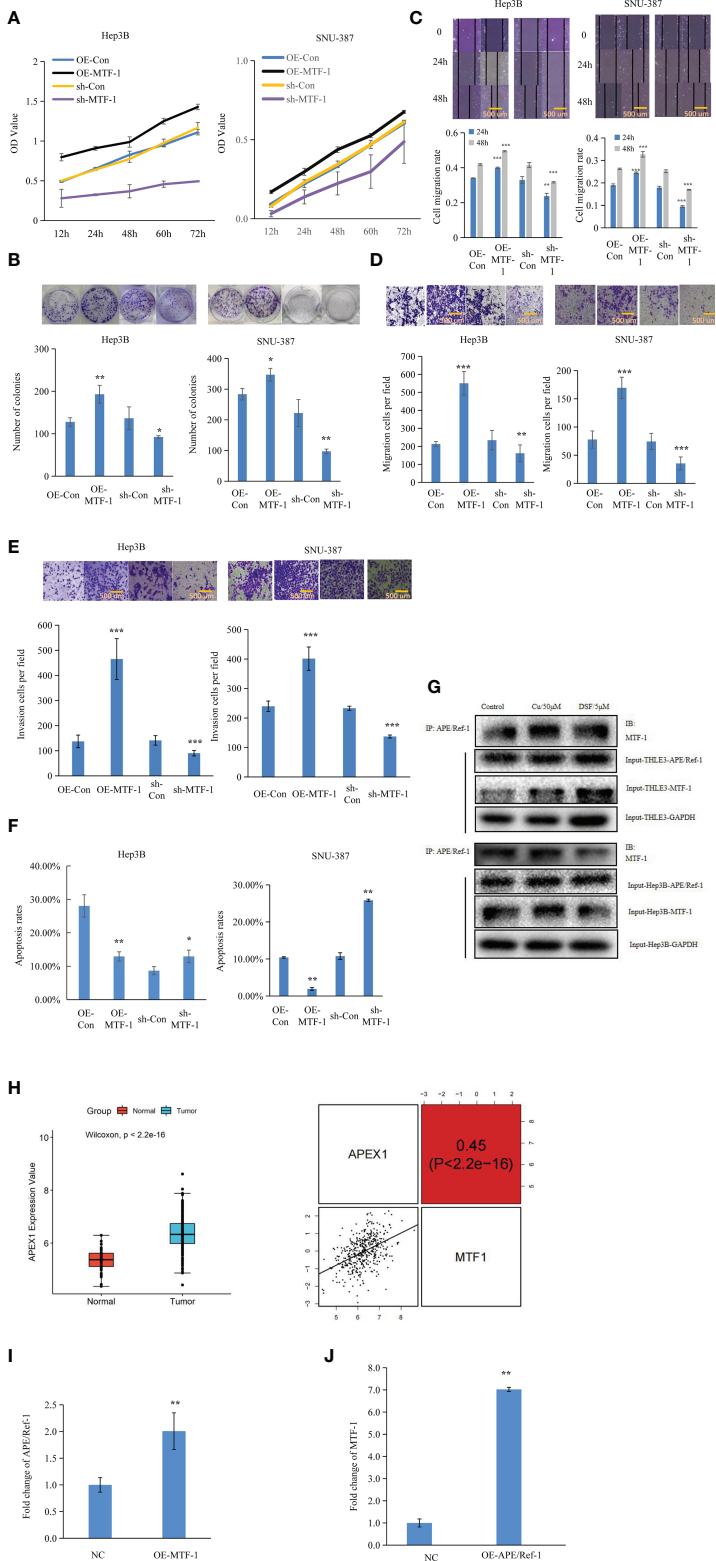
Metal-regulatory transcription factor-1 (MTF-1) promoted cell proliferation, migration, and invasion as a key mediator and regulator. The regulatory roles of MTF-1 in cell proliferation were evaluated using the 3-(4,5-dimethylthiazol-2-yl)-2,5-diphenyltetrazolium bromide (MTT) and colony formation assays **(A, B)**. Effect of MTF-1 on cell motility was assessed using the wound healing assay. Scale bar = 500 μm **(C)**. MTF-1-promoted hepatocellular carcinoma (HCC) cell migration and invasion, as determined using the Transwell assay. Scale bar = 500 μm **(D, E)**. Cell apoptotic rates were detected after different treatments **(F)**. Co-immunoprecipitation (Co-IP) study showed specific binding between MTF-1 and APE/Ref-1 with different treatments **(G)**. MTF-1 expression positively correlated with upregulated APE/Ref-1 level, as determined using the integrative analysis of The Cancer Genome Atlas (TCGA) data **(H)**. Overexpression of MTF-1 activated APE/Ref-1, which in turn initiated the upregulation of MTF-1 level in HCC cells **(I, J)**. Data are presented as mean ± SD. *p < 0.05, **p < 0.01, ***p < 0.001.

To further elucidate the role of MTF-1 in HCC progression, the effects of MTF-1 knockdown and overexpression were analyzed. The MTT and colony formation assays showed that activated MTF-1 promoted cell proliferation, which was consistent with reduced cell viability after the knockdown of MTF-1 in HCC (**Figures 2A, B**). As shown in **Figures 2C–F**, MTF-1 stimulated cell migration and invasion, and enhanced the anti-apoptotic capability of HCC cells. In particular, MTF-1 showed co-immunoprecipitation (co-IP) with APE/Ref-1 (**Figure 2G**, **Supplementary Figure S2**), highlighting the specific binding between MTF-1 and APE/Ref-1, which is implicated in HCC progression (14). The integrative analysis of TCGA data also indicated that upregulated APE/Ref-1 expression positively correlated with MTF-1 expression (**Figure 2H**). The overexpression of MTF-1 activated APE/Ref-1, which, in turn, upregulated MTF-1 expression in HCC (**Figures 2I, J**). These findings strengthen the hypothesis that MTF-1 potentiates HCC progression by serving as a key regulator and mediator.

### MTF-1 Is a Direct Target of miR-148-3p

**Figure 3:**
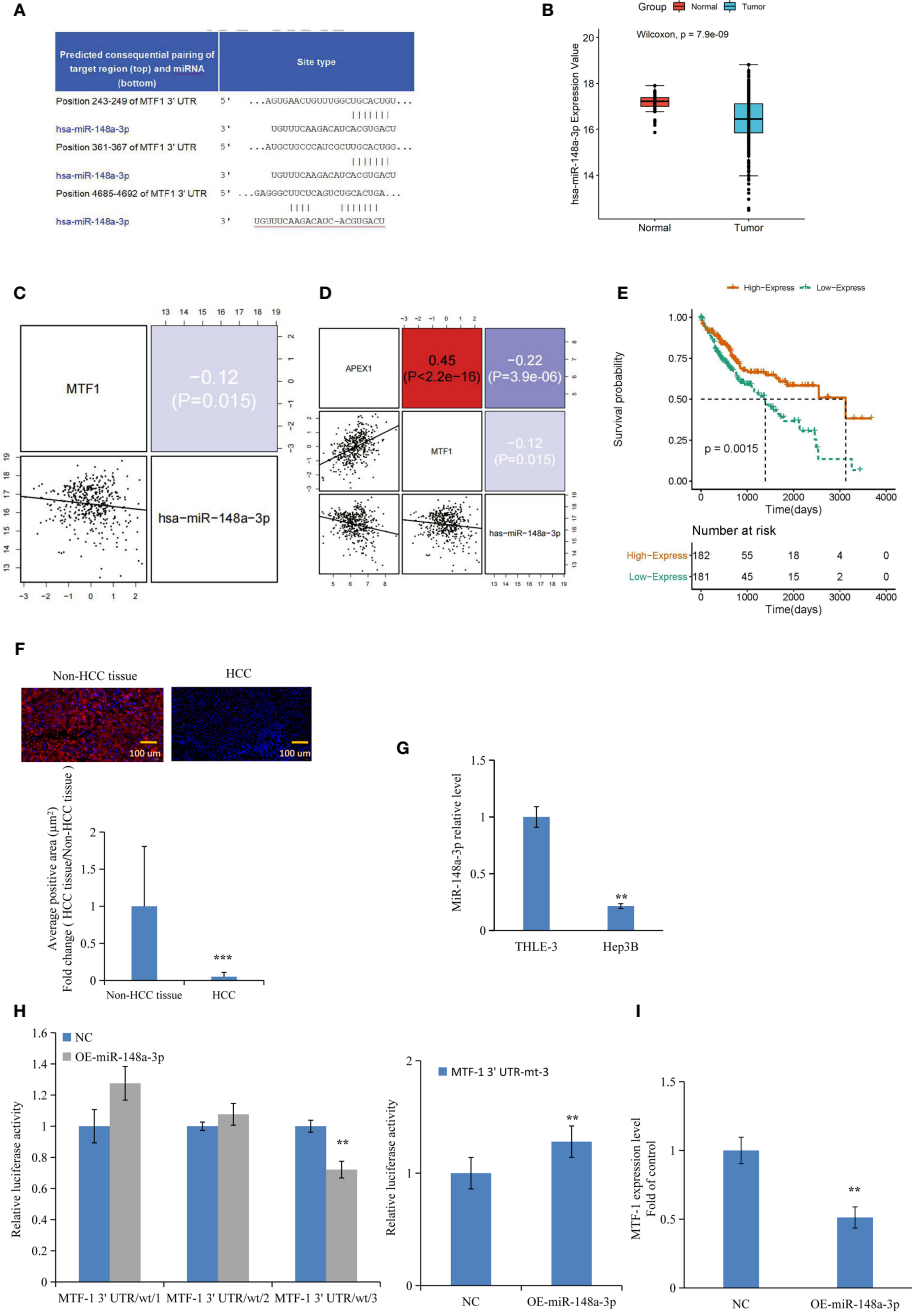
Metal-regulatory transcription factor-1 (MTF-1) is directly targeted by miR-148a-3p. The predicted binding sites of miR-148a-3p in the 3′-UTR of MTF-1 were determined **(A)**. Integrative analysis of The Cancer Genome Atlas (TCGA) data indicated that miR-148a-3p expression was downregulated in tumors **(B)**, and inversely correlated with MTF-1 and APE/Ref-1 levels **(C, D)**. Strong miR-148a-3p expression was associated with improved survival **(E)**. MiR-148a-3p level was assessed in tissue microarrays (TMAs) with considerably reduced average positive area in hepatocellular carcinoma (HCC) tissue. Scale bar = 100 μm **(F)**. Downregulated miR-148a-3p expression was observed in HCC cells **(G)**. Overexpression of miR-148a-3p reduced luciferase activity of MTF-1-wt (wild-type) instead of MTF-1-mt (mutant-type) **(H)**. MiR-148a-3p overexpression inhibited MTF-1 expression in HCC cells **(I)**. Data are expressed as mean ± SD. **p < 0.01, ***p < 0.001.

**Figure 4:**
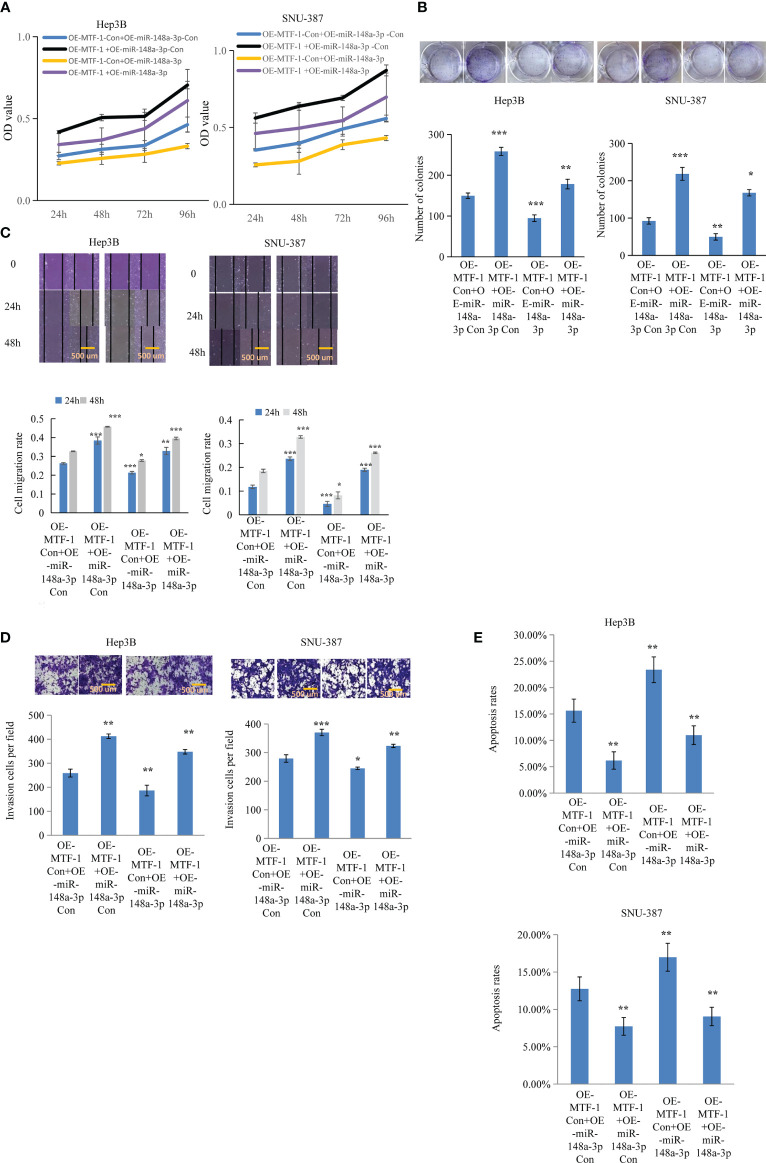
MiR-148a-3p overexpression reversed the effects of activated metal-regulatory transcription factor-1 (MTF-1) in hepatocellular carcinoma (HCC) cells. To further elucidate the regulatory role of miR-148a-3p, Hep3B and SNU-387 cells were treated with or without miR-148a-3p mimics after MTF-1 overexpression. miR-148a-3p overexpression reversed the effects of activated MTF-1 in HCC cell proliferation **(A)**, colony formation **(B)**, migration **(C)**, invasion **(D)**, and apoptosis **(E)**. Scale bar = 500 μm. Data are expressed as mean± SD. *p < 0.05, **p < 0.01, ***p < 0.001.

**Figure 5:**
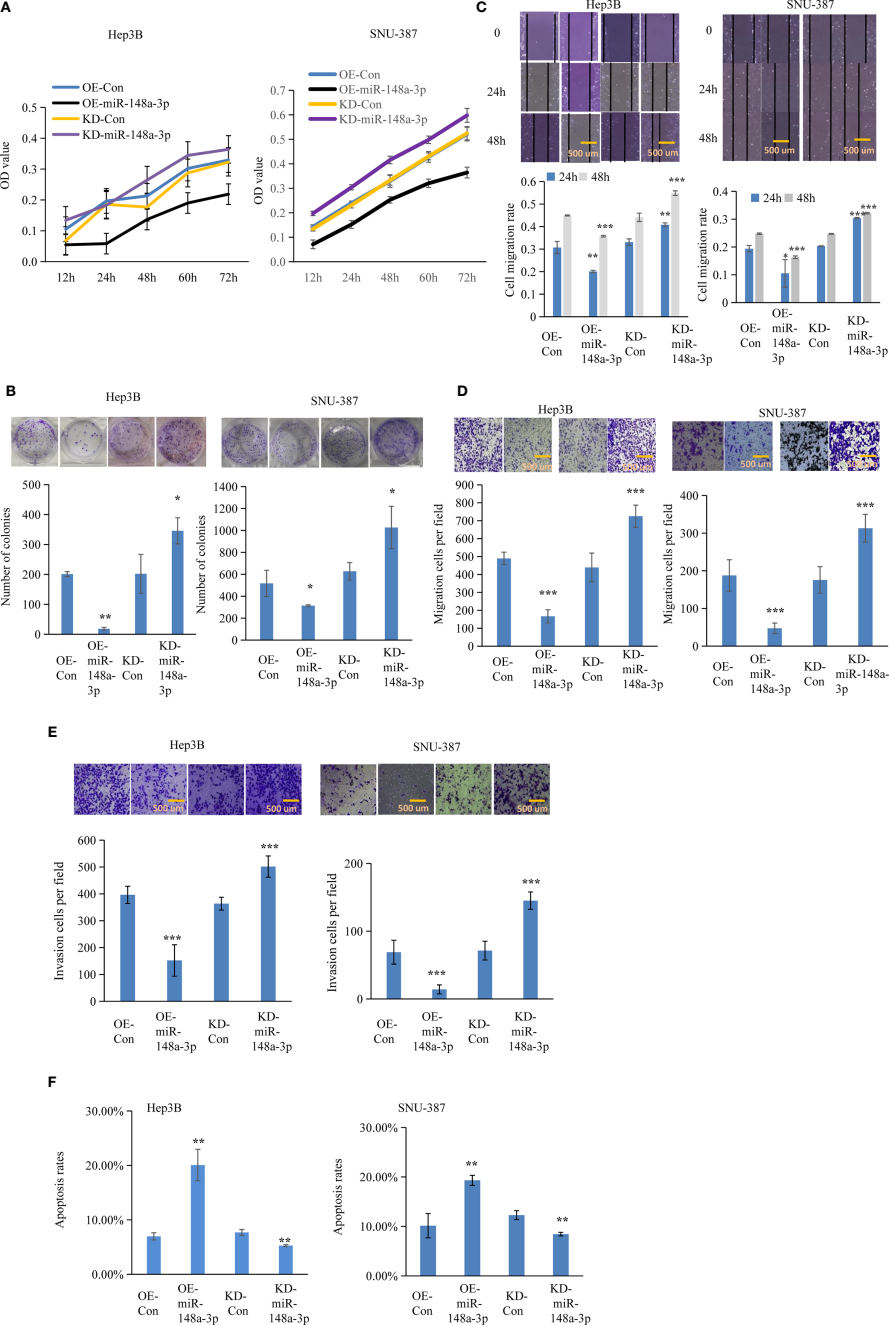
Function of miR-148a-3p on cell viability, colony formation, and metastasis. The effect of miR-148a-3p alone was further evaluated. Capacities of cell proliferation **(A)**, colony formation **(B)**, migration **(C, D)**, and metastasis **(E)** were suppressed with miR-148a-3p overexpression; miR-148a-3p knockdown facilitated cell mobility and invasion, and enhanced the anti-apoptotic capability **(F)** in hepatocellular carcinoma (HCC) cells. Scale bar = 500 μm. Data are expressed as mean ± SD. *p < 0.05, **p < 0.01, ***p < 0.001.

**Figure 6:**
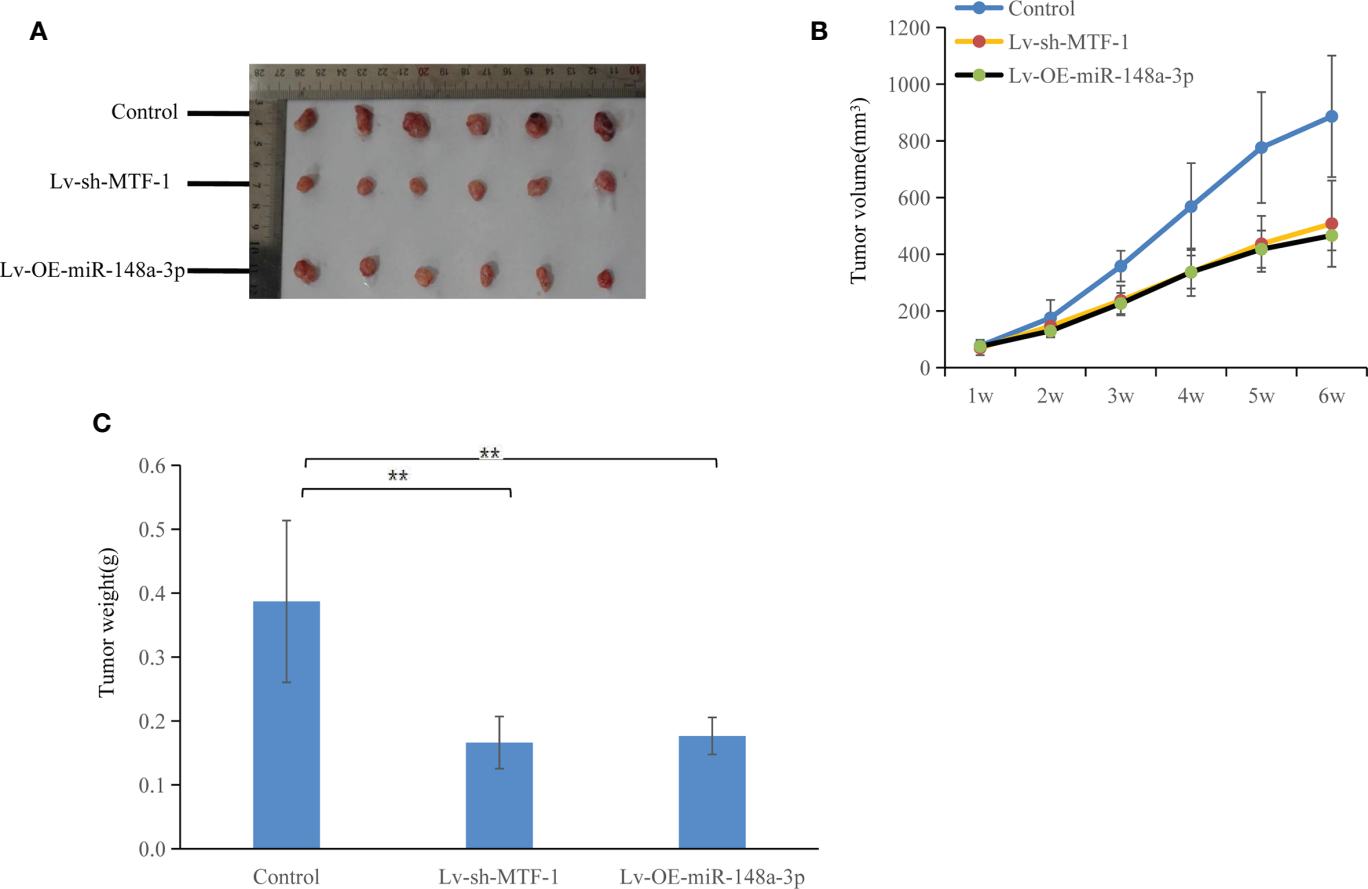
Both metal-regulatory transcription factor-1 (MTF-1) knockdown and miR-148a-3p-overexpression suppressed tumor growth. *In vivo* experiments were conducted using a nude mouse subcutaneous xenograft model: slow growth of MTF-1-knockdown or miR-148a-3p-overexpressing Hep3B-derived xenografts was indicated **(A)**, with reduced tumor volume **(B)** and weight **(C)** compared with the control group. Significant difference was observed in tumor growth and weight among the three groups. Data are expressed as mean ± SD. **p < 0.01.

Based on the analysis of databases miRDB (http://www.mirdb.org/) and miRanda (http://www.microrna.org), we predicted that miR-148a-3p and MTF-1 sequences were complimentary to each other (**Figure 3A**). Furthermore, TCGA data analysis showed the downregulation of miR-148a-3p in tumors and its inverse correlation with MTF-1 and APE/Ref-1 expression (**Figures 3B–D**). Notably, a strong miR-148a-3p expression was associated with improved survival of patients (**Figure 3E**). Subsequently, the miR-148a-3p level was detected in TMA and HCC cells. Normal tissue presented a significantly higher miR-148a-3p level than the HCC tissue (**Figure 3F**) (*p* < 0.001), suggesting that miR-148a-3p expression and its tumor-suppressive activity might be lost in tumor tissues. Correspondingly, a decreased level of miR-148a-3p was also established in HCC cells (**Figure 3G**). For further analysis, we carried out dual-luciferase reporter assay and RT-PCR. The overexpression of miR-148a-3p considerably reduced the luciferase activity of MTF-1-wt-3 (wild type). However, the luciferase activity of MTF-1-mt (mutant type) cells transfected with miR-148a-3p mimic (**Figure 3H**) was not inhibited, indicating that miR-148a-3p might directly target MTF-1. The overexpression of miR-148a-3p inhibited MTF-1 expression in HCC cells (**Figure 3I**). Moreover, miR-148a-3p overexpression also reversed the effects of activated MTF-1 on cell growth, migration, and invasion (**Figures 4A–E**). In addition, cell viability, colony formation, and metastasis were affected by miR-148a-3p alone; treatment with miR-148a-3p knockdown facilitated cell migration and invasion, and enhanced the anti-apoptotic ability in HCC cells (**Figures 5A–F**). To further establish the above findings, *in vivo* experiments were performed on a xenograft mouse model. MTF-1-knockdown and miR-148a-3p-overexpressing Hep3B-derived xenografts showed a slower growth rate, with reduced tumor volume and weight compared with the control group (**Figures 6A–C**). There was a significant difference in tumor growth and weight among the three groups. Thus, MTF-1 was directly targeted by miR-148a-3p, and the miR-148a-3p/MTF-1 axis could be of significance in HCC progression.

### miR-148-3p-Containing Exosomes Inhibit HCC Cell Proliferation and Metastasis by Inhibiting MTF-1

**Figure 7:**
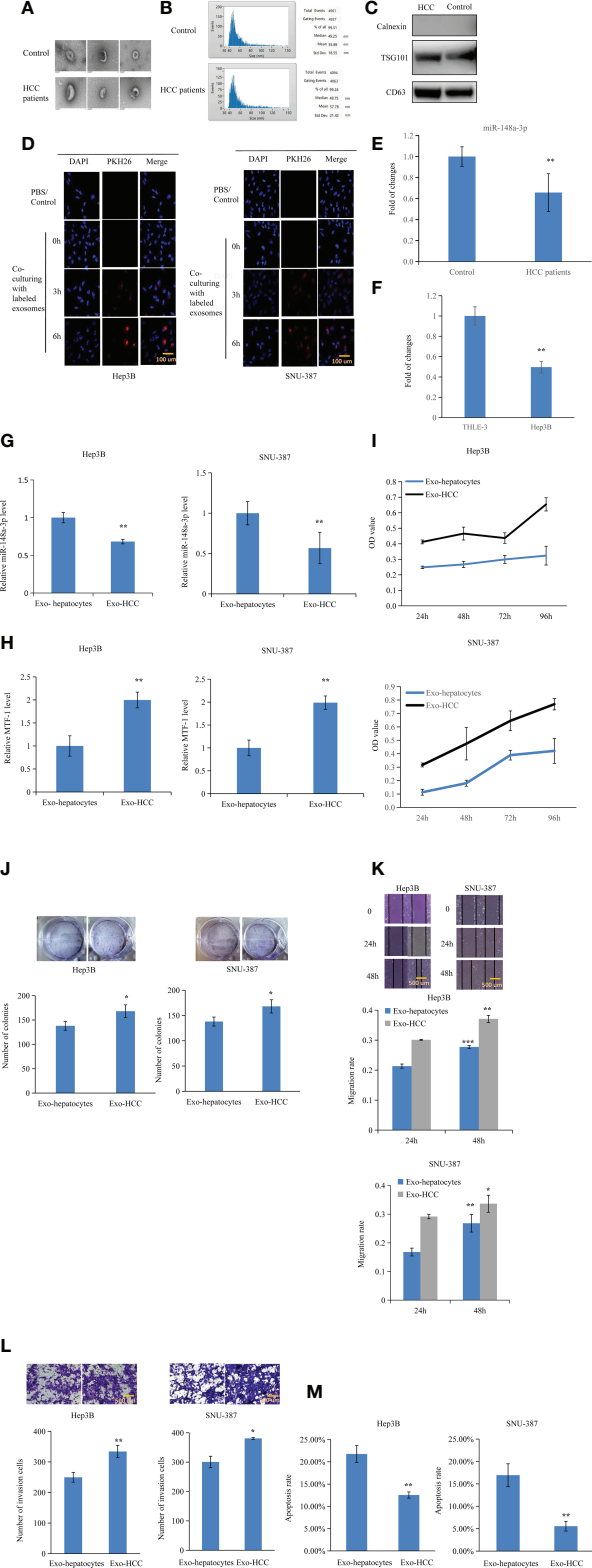
Inhibitory effects of exosomal miR-148a-3p on hepatocellular carcinoma (HCC) cells. Exosomes were isolated and RNA was extracted. The morphology of exosomes was evaluated by transmission electron microscopy **(A)**. Size distribution of exosomes was verified **(B)**. Western blotting was performed to identify exosome markers **(C)**. Exosomes labeled with PKH-26 red fluorescent dye were observed in Hep3B and SNU-387 cells after co-incubation. Scale bar = 100 μm **(D)**. miR-148a-3p was downregulated in the exosomes isolated from the plasma of patients with HCC and culture media of HCC cells **(E, F)**. To further elucidate the role of exosomal miR-148a-3p, miR-148a-3p and metal-regulatory transcription factor-1 (MTF-1) levels were evaluated after culturing with exosomes from hepatocyte- or HCC-conditioned medium **(G, H)**. HCC cell proliferation, mobility, and invasion were also inhibited in the presence of exosomes isolated from hepatocyte-conditioned medium. Scale bar = 500 μm **(I–L)**. Co-culture with exosomes from hepatocyte- conditioned medium induced HCC cell apoptosis **(M)**. Data are expressed as mean ± SD. *p < 0.05, **p < 0.01, ***p < 0.001.

Exosomes were isolated and RNA was extracted for further analysis. The morphology of exosomes was verified by transmission electron microscopy (**Figure 7A**). Size distribution of exosomes was also analyzed (**Figure 7B**). Exosome markers were established by Western blotting (**Figure 7C**). In addition, exosomes were labeled with PKH-26 red fluorescent dye and the uptake of labeled exosomes was observed and verified (**Figure 7D**). miR-148a-3p expression decreased in the exosomes isolated from the serum of patients with HCC and from the medium of HCC cells (**Figures 7E, F**). To further clarify the effect of exosomal miR-148a-3p on target protein and cells, we isolated exosomes from hepatocyte- or HCC-conditioned medium and used them to treat HCC cells. When HCC cells were cultured with exosomes from hepatocytes, higher miR-148a-3p and lower MTF-1 levels were observed in the qPCR analysis (**Figures 7G, H**). As expected, weakened cell viability, migration, and invasion, and induced apoptosis were observed in the presence of exosomes isolated from hepatocyte-conditioned medium (**Figures 7I–M**). Collectively, these results indicated that exosomal miRNAs could be transferred into the tumor microenvironment, and exosomal miR-148a-3p might also play an important role. A further investigation is necessary to evaluate the potential role of exosomal miR-148a-3p/MTF-1 as an effective target for HCC treatment.

## Discussion

In 2020, there were 19.3 million new cases of cancer and 10.0 million cancer-related deaths. In particular, HCC has been projected as one of the top three cancers to cause cancer-related deaths by 2030. Although many new therapeutics have been approved for clinical application, the survival rate of patients with HCC has not considerably improved over the past two decades (15, 16). Understanding the mechanisms controlling HCC progression in detail is of importance to develop new therapeutic approaches.

Accumulating evidence has demonstrated that an increase in copper level is closely associated with cancer progression. Copper accumulation in Long-Evans Cinnamon rat livers leads to spontaneous HCC (17). Conversely, copper-depleted animals have relatively avascular tumors and decreased invasive capacity (18). In our previous study, we found that copper induced hepatocyte proliferation and activated signaling involved in HCC genesis and progression (14). In particular, metal homeostasis and detoxification are generally controlled transcriptionally by metal-sensing signals through proteins such as metallothioneins (MTs; MT1 and MT2), which are modulated by MTF-1 (19, 20). MTF-1 has N- and C-terminal regions with a modular transcription activation domain, and six zinc fingers forming the DNA-binding domain. Heavy metals, redox stress, growth factors, and cytokines induce MTF-1 expression. This protein is structurally and functionally conserved, indicating that it plays an important role in maintaining metal homeostasis across species (21–23). The knockout of *MTF-1* in mice resulted in impaired liver development and led to liver decay and embryonic death, suggesting a critical role of MTF-1 in liver-specific developmental gene expression (24). However, in mammals, MTF-1 may have more complicated biological functions. In particular, more attention has been focused on its role in tumorigenesis and disease progression. In the present study, MTF-1 expression was upregulated in HCC cells and tissue. Furthermore, a higher expression of MTF-1 was associated with poor survival and recurrence, suggesting that MTF-1 accumulation is closely related to the clinical outcome as an independent risk factor of HCC recurrence and mortality. To further clarify the role of MTF-1 in HCC progression, we performed cell viability, colony formation, wound healing, Transwell assays, and flow cytometry analysis. As expected, MTF-1 promoted cell proliferation, stimulated cell migration and invasion, and enhanced anti-apoptotic capability of HCC. Hence, MTF-1 might be involved in HCC genesis and development as a regulating factor. Consequently, it is necessary to assess other factors that mediate or interact with MTF-1 in HCC. APE/Ref-1 is a potential candidate, which is an important mediator and potentiator of HCC progression (14). APE/Ref-1 is a master regulator of cellular responses to oxidative stress and affects tumor progression by transactivating numerous transcription factors, which have been implicated in cell proliferation and metastasis. Interestingly, the co-IP study showed specific binding between MTF-1 and APE/Ref-1. Subsequent analysis revealed that MTF-1 expression positively correlated with the APE/Ref-1 level. In addition, the overexpression of MTF-1 activated APE/Ref-1, which in turn upregulated MTF-1 expression in HCC. Thus, MTF-1 and APE/Ref-1 may act in concert in HCC progression.

Exosomes are extracellular vesicles and are widely present in various body fluids. Cancer cells secrete at least 10-fold more exosomes than normal cells, and tumor-derived exosomes could promote cell–cell communication through the transport of miRNAs and other small molecules (25). Recently, miRNAs have been identified in exosomes modulating cell proliferation, migration, and metastasis (26). miRNAs are a major class of small non-coding RNAs that mediate post-transcriptional gene silencing by binding to the 3′-UTR or ORFs of target mRNAs. Exosomes protect miRNAs from degradation and then facilitate them to be stably expressed in the extracellular space and be efficiently integrated by specific recipient cells (27). Recently, emerging evidence demonstrates that exosomal miRNAs regulate cancer progression. Here, we collected exosomes from the plasma of patients with HCC and culture supernatant of HCC cells and found that miR-148a-3p expression was significantly downregulated. In addition, our findings also revealed that miR-148a-3p expression decreased in both HCC tissue and cells. MTF-1 is a direct target of miR-148a-3p. Correspondingly, miR-148a-3p overexpression inhibited MTF-1 expression and reversed the effects of activated MTF-1 in HCC cells. To delineate the effects of exosomal miR-148a-3p on target protein and cells, we isolated exosomes from hepatocyte- or HCC-conditioned medium and treated HCC cells. The qPCR analysis revealed higher miR-148a-3p and lower MTF-1 expressions in the presence of exosomes from hepatocyte culture media as well as weakened cell viability and migration, and enhanced apoptosis. Notably, stable MTF-1-knockdown or miR-148a-3p-overexpressing Hep3B-derived xenografts grew at a slower rate with reduced tumor volume and weight than the control group. Therefore, these results suggest that exosomal miR-148a-3p was transferred in the HCC microenvironment and the exosomal miR-148a-3p/MTF-1 axis should be of importance in HCC progression.

In conclusion, MTF-1 is a potential therapeutic target for manipulating metal and/or redox homeostasis in HCC. Additionally, this study provides a basis for further investigation utilizing appropriate MTF-1/APE/Ref-1 inhibitors in combination with chemotherapeutics for HCC treatment. Exosomal miR-148a-3p is also a promising non-invasive biomarker and a potential target factor in HCC diagnosis and treatment. However, in depth studies would be necessary to identify other mechanisms that may be involved in the regulation of MTF-1 and the potential correlation between APE/Ref-1 and miR-148a-3p.

## Data Availability Statement

The raw data supporting the conclusions of this article will be made available by the authors, without undue reservation.

## Ethics Statement

The studies involving human participants were reviewed and approved by the Medical Ethics Committee of Shandong Provincial Hospital and the Medical Ethics Committee of Shanghai Outdo Biotech Company. The patients/participants provided their written informed consent to participate in this study. The animal study was reviewed and approved by the Animal Ethics Committee of Shandong Provincial Hospital.

## Author Contributions

ZY designed the study. ZL, MY, TY, MM, and ZY collected and analyzed the results. ZY wrote the first draft of the manuscript. All authors contributed to manuscript revision. All authors contributed to the article and approved the submitted version.

## Funding

This research was supported by the National Natural Science Foundation of China (No. 81972606) and Foundation of Shandong Natural Science Foundation (ZR2019MH005).

## Conflict of Interest

The authors declare that the research was conducted in the absence of any commercial or financial relationships that could be construed as a potential conflict of interest.

## Publisher’s Note

All claims expressed in this article are solely those of the authors and do not necessarily represent those of their affiliated organizations, or those of the publisher, the editors and the reviewers. Any product that may be evaluated in this article, or claim that may be made by its manufacturer, is not guaranteed or endorsed by the publisher.
