# Supplementary figures and images for: Metal-Regulatory Transcription Factor-1 Targeted by miR-148a-3p Is Implicated in Human Hepatocellular Carcinoma Progression
Source: Front Oncol. 2021 Sep 29;11:700649. doi: 10.3389/fonc.2021.700649 (PMC8511627; doi:10.3389/fonc.2021.700649)

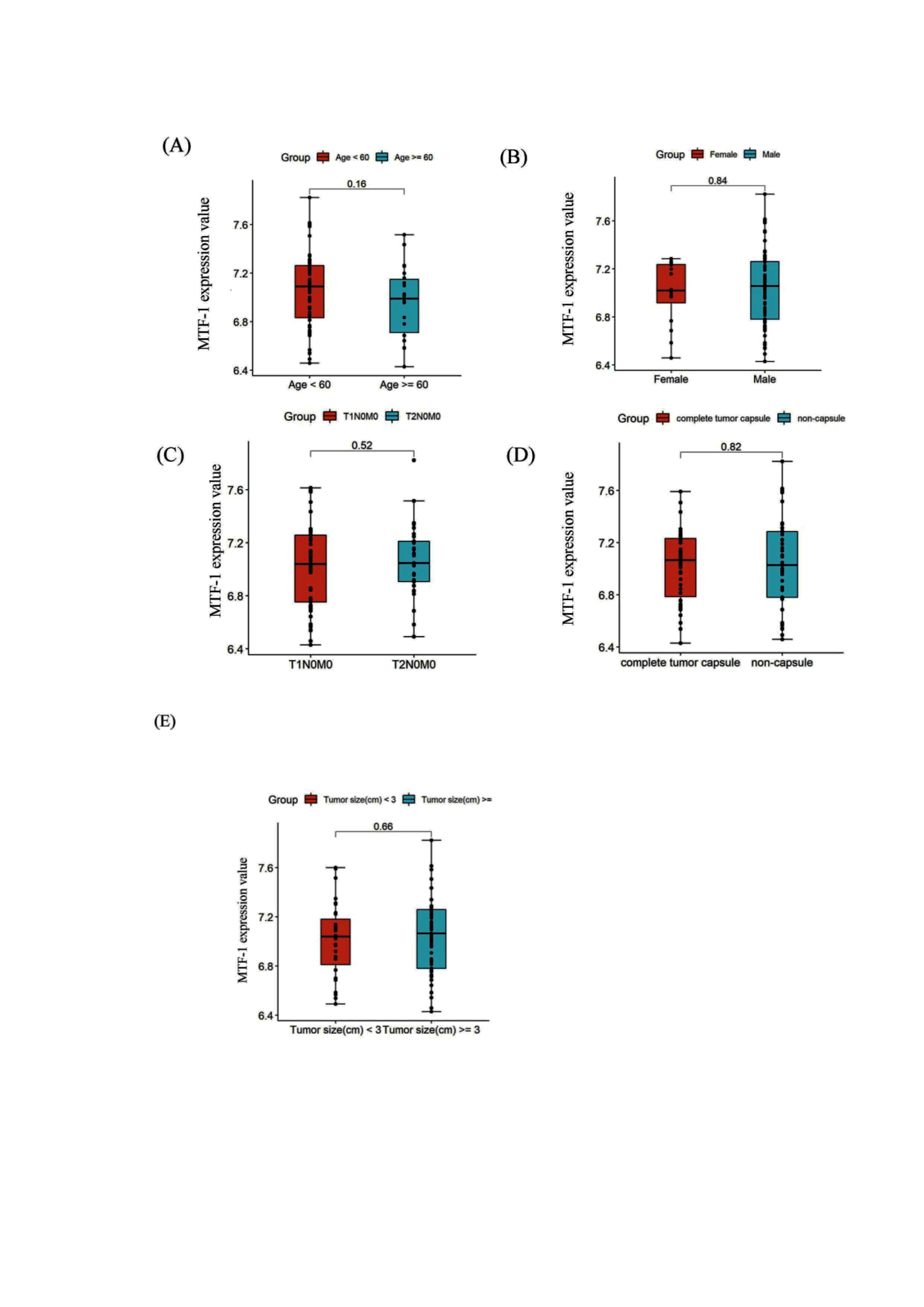

Supplement: Supplementary file 1 [file Image_1.jpeg]

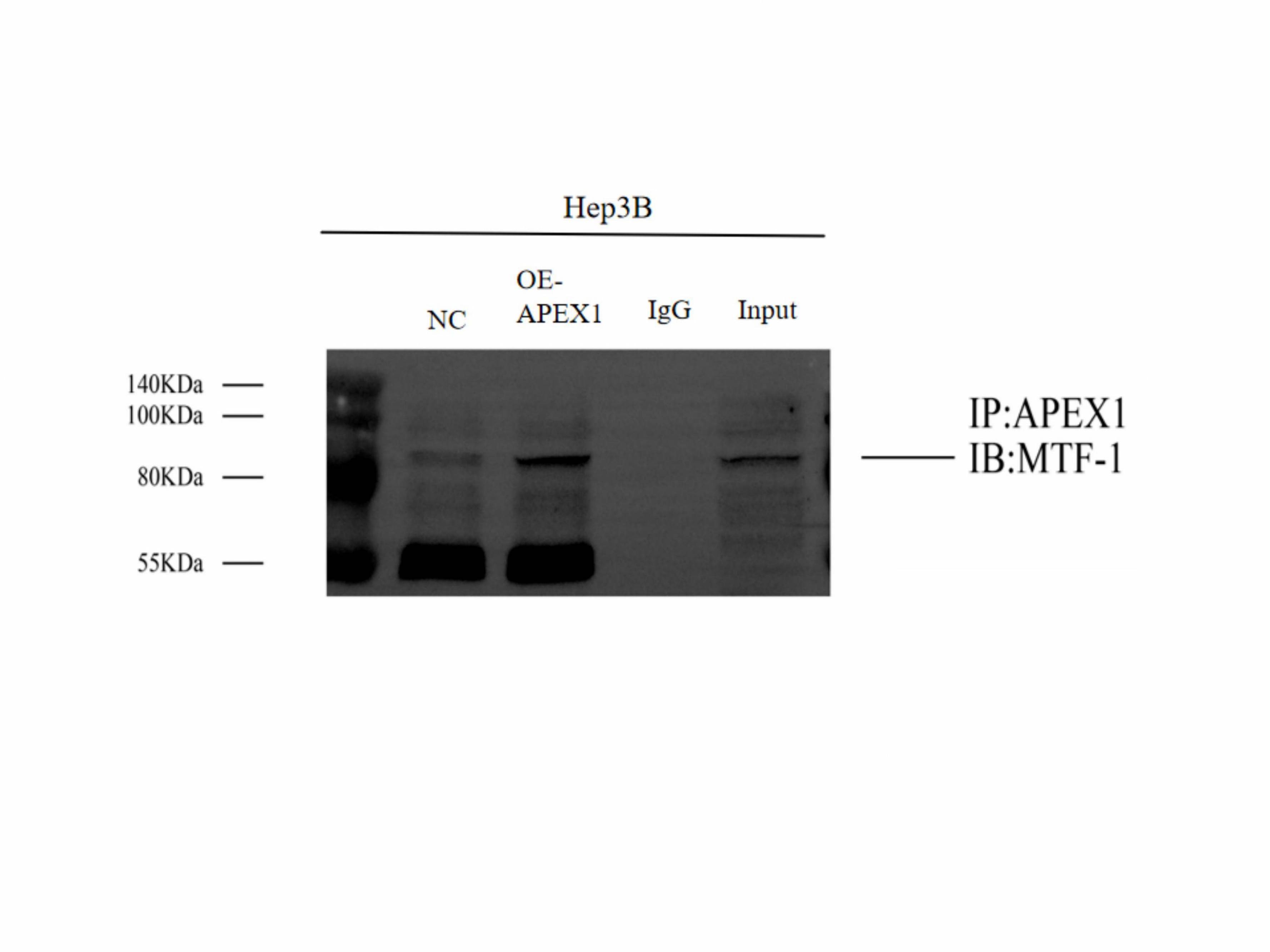

Supplement: Supplementary file 2 [file Image_2.jpeg]
